# Perceptions of Rule-Breaking Related to Marine Ecosystem Health

**DOI:** 10.1371/journal.pone.0089156

**Published:** 2014-02-27

**Authors:** Matthew J. Slater, Yunus D. Mgaya, Selina M. Stead

**Affiliations:** 1 School of Marine Science and Technology, Newcastle University, Newcastle upon Tyne, Tyne and Wear, United Kingdom; 2 Department of Aquatic Sciences and Fisheries, University of Dar es Salaam, Dar es Salaam, Tanzania; Aristotle University of Thessaloniki, Greece

## Abstract

Finding effective solutions to manage marine resources is high on political and conservation agendas worldwide. This is made more urgent by the rate of increase in the human population and concomitant resource pressures in coastal areas. This paper links empirical socio-economic data about perceptions of marine resource health to the breaking of marine management rules, using fisheries as a case study. The relationship between perceived rule-breaking (non-compliance with regulations controlling fishing) and perceived health of inshore marine environments was investigated through face-to-face interviews with 299 heads of households in three Tanzanian coastal communities in November and December 2011. Awareness of rules controlling fishing activity was high among all respondents. Fishers were able to describe more specific rules controlling fishing practices than non-fishers (t = 3.5, df = 297, p<0.01). Perceived breaking of fishing regulations was reported by nearly half of all respondents, saying “some” (32% of responses) or “most” (15% of responses) people break fishing rules. Ordinal regression modelling revealed a significant linkage (z = −3.44, p<0.001) in the relationship between respondents' perceptions of deteriorating marine health and their perception of increased rule-breaking. In this paper, inferences from an empirical study are used to identify and argue the potential for using perceptions of ecosystem health and level of rule-breaking as a means to guide management measures. When considering different management options (e.g. Marine Protected Areas), policy makers are advised to take account of and utilise likely egoistic or altruistic decision-making factors used by fishers to determine their marine activities.

## Introduction

Rule-breaking by resource users is a common explanation for fisheries management failing to achieve sustainability and conservation goals worldwide. An increasing amount of information shows significant and negative impacts of fishers' rule-breaking on fish stocks, national economies and global trade [1]. However, finding solutions to achieve compliance to support sustainable marine resources management remains a global unresolved challenge. Understanding the reasoning, perceptions, pressures and socio-economic drivers behind decisions made by fishers, at all scales, to break fishing rules remains central to achieving compliance and viable fish stock management [2], [3]. Understanding and integrating the context-specific characteristics of rule-breaking allows management measures to be tailored to address rather than compound local issues.

Research to date has attempted to describe rule-breaking's justification through moral objections or through fisher's reasoned decisions to comply or not. In the first instance rule-breaking by fishers is favoured by their view that rules are unfair or illogical, that rules are contrary to their conscience, or that rule-breaking is morally acceptable due to higher needs, for example, one must catch fish by any means in order to feed one's family [4]. Equally, fishers may break rules set by authorities they do not respect or accept as ruling their fishing areas (lack of co-governance). Yet, despite the agreed importance of belief systems and moral aspects in rule-breaking, it is most commonly argued that fisheries rule-breaking stems from simple economic cost∶benefit analysis [1], [4]. Using similar assumptions to rational choice theory in criminology, Sumaila et al [1] argue that fishers decide as individuals in a calculated manner whether it fulfils their immediate economic needs and is worthwhile to risk breaking the rules controlling fishing [5]. When applying this model, fishers may decide whether the benefits of increased catch and/or reduced catch effort obtained through breaking rules controlling fishing will be greater than: 1) cost of being caught (fines/confiscation), including an inherent calculation of likelihood of being caught and cost of avoidance behaviour required; 2) perceived benefit of complying; 3) social implications of fisher's involvement in rule-breaking. Sumaila et al. [1], argue that in most cases this cost∶benefit analysis favours the decision to break rules.

If these assumptions are correct, the above model predicts a global economic weighting towards rule-breaking which can be explained by the relatively low chances of being caught breaking rules due to the geographical size of marine areas required to be monitored, insufficient resources to enforce rules and lack of economic viability of enforcement [6]. While enforcement can be effective in smaller defined marine areas, such as no-take zones or small marine protected areas, most nations have insufficient resources and capacity to effectively enforce fishing laws over the majority of their territorial waters [1], [7].

Increasingly draconian and complex laws along with heavy punishments also generally lead to a continuous cycle of increased rule-breaking among fishers due to confusion, resentment and loss of respect for rules [8]. Furthermore, there exists a lack of self–enforcement of fishing rules, i.e. fishermen are generally unlikely to inform on their rule-breaking colleagues. Commonly, with external actors unable to enforce rules, and a lack of enforcement by other fishers, individual fishers are solely responsible for deciding if a rule will be broken [8]. Thus studying socio-economic drivers that underpin individual decision-making behaviour can identify a range of factors which may be linked to likelihood of rule-breaking under different local conditions. This information can be applied to a wide range of marine management scenarios globally as part of using context-specific knowledge to promote and gain support for specific rules.

Where enforcement is ineffective or of secondary importance, individual fisher's compliance can be dependent on personal conviction on the one hand and on societal expectation on the other. Fishers may be more likely to comply if they feel social pressure or moral obligation to accept them [2], [9]. If community and individual/household conditions are appropriate, then perceived and actual involvement of individual fishers in rule-making and management (i.e. co-management and co-enforcement) can be effective in making rules more logical and morally acceptable to fishers, and also increases awareness of rules [10]. Co-management has been credited with supporting positive effects in compliance within coastal resource management programmes worldwide, despite the measures taken often offering no economic benefits on the fishers who support them [11], [12].

In the current research we argue, individual perceptions (what individuals perceive or believe they perceive as states, such as marine ecosystem health, or occurrences) while representative and subjective, can influence reasoning and ultimately behavioural choices. To illustrate, an awareness and acceptance of the problem related to a specific rule (in this case health of a threatened marine fish stock) has been shown to have a positive effect on willingness to comply with that rule, although the underlying stimuli can be complex [13], [14]. Jager et al. [4] showed that concern about levels of fish depletion increased fishers' belief that rule-breaking was unacceptable in the case of fishing for food, although this did not extend to the fishers own target species when examined in view of expected fishing income from commercial stocks. Fishers' acceptance of a specific environmental problem or overall stock degradation and the clearly communicated association of that problem with fishing rules intended to counter it may increase individual willingness to comply. It is unclear whether this applies at a societal level, resulting in pressure or expectation to comply.

Effective fisheries management, including clear communication about enforcement efforts and regulatory measures can benefit from being formed on the basis of information about how fishers and societies they belong to perceive rule-breaking. The current study aims to contribute to understanding of the reasoning, perceptions and socio-economic drivers underlying fishers' decision-making about how they fish, specifically about breaking of fishing rules.

This paper reports the findings from interviews with 299 heads of households in three fishing communities in the United Republic of Tanzania. The perceptions of respondents, both fishers and non- fishers, within fishing communities, that is, the society fishers belong to, are analysed specifically to isolate the social variables statistically linked to perceived rule-breaking. Identifying relevant perceptions and associations to compliance allows a discussion of potential effects on behavioural choice. From a proactive management stance this knowledge can be used to map out scenarios to be presented to local communities to communicate possible consequences of their actions. This can allow informed choice, for example, about which conservation measures are more likely to have favourable outcomes and thus be supported. The overall aim is to understand relevant socio-economic variables that may find application in improving fisheries management, thus supporting recommendations for future use in management options and policy making.

## Methods

### 2.1 Ethics statement

Ethics approval was sought and obtained for this study through submission of an ethics assessment to the Newcastle University Ethical Committee by which all methods including verbal consent were deemed acceptable and of no ethical concern. Researchers obtained verbal consent from participants before conducting household surveys. In this case consent was documented only in completion of the interview cover sheet (i.e. a survey ID was assigned and the interviewer recorded their own name and began interviewing). The verbal consent procedure ensured (as a minimum) participants were informed about the survey, its purpose, and how the data would be utilized and were informed they would receive reports on findings at community presentations. Written consent was not sought as it was possible that the low literacy level of a number of respondents would have made written consent unrepresentative of actual consent. Participant's names were not recorded nor were other personal details which could be used to identify individual respondents.

### 2.2 Survey design

**Figure 1:**
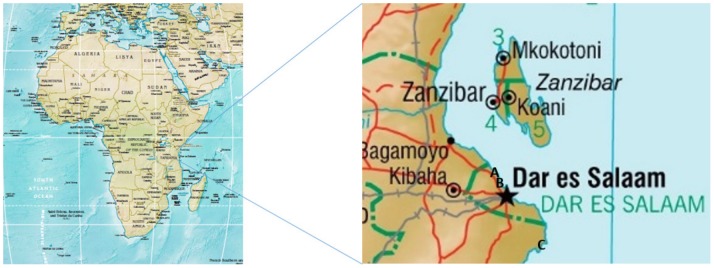
Map of Tanzania with expanded area central coast of Tanzania indicating position of coastal villages from North to South: A. Ununio, B. Kunduchi, and C. Buyuni. (Source https://www.cia.gov/library/publications/cia-maps-publications/Tanzania.html).

Face-to-face interviews were conducted with 299 heads of households in three coastal villages - Ununio, Kunduchi and Buyuni -in the United Republic of Tanzania in November and December 2011 (Figure 1). Study sites varied according to economic status, proximity to the coast and dependence on small-scale fisheries. To summarise:

- Ununio is 20 kilometres north of Dar-es-Salaam (estimated population 1050; 168 households).
- Kunduchi is 15 km north of Dar-es-Salaam (estimated population 1370; 212 households).
- Buyuni is 50 km south of Dar-es-Salaam, (estimated population 1100; 204 households).

Ununio and Kunduchi are (‘diffuse’) peri-urban fishing villages on the outskirts of Dar es Salaam, with encroachment of urban expansion on land area for agriculture and construction [15]. Buyuni is rural, and requires 3–4 hours car travel to reach from Dar es Salaam. There is no known history of co-management or participation in rule-making within the three fishing villages, there is however a no-take Marine Protected Area on Mbudya Island in close proximity to Kunduchi which is partially operated by ex-fishers.

Villages were mapped using satellite images as a reference and ground-proofed by in-field observations to create full hand-drawn maps of all roads, pathways and dwellings. Maps were then used to systematically sample (numbered) households within each community. The head of household of every second house was interviewed.

‘Household’ was defined as a unit of people that share a house [16]. Heads of households were interviewed where possible as decision-makers considered to hold more detailed information about current livelihoods, income and socio-economic characteristics. If the head of household was not available at the second visit, interviewers requested to interview an adult from the household fully informed about the household's full range of income-related activities and livelihoods.

Data collected from the interviews described respondents' dependence on marine resources and perceptions of marine health. Respondents discussed their current economic status and employment along with awareness of rules to control fishing activity (open) and were asked questions focussed on whether people break these rules. Response options were not prescriptive or numerical but were designed to provide a simple ordinal-scale estimate of perceived levels of rule-breaking. Thus response options to the question “Do people break these rules?” were: Yes, most people break them; Yes, some people break them; Yes, but very few people break them; No, no-one breaks them.

Household income, possessions and utilities, such as electricity, mobile phone and other physical assets such as house construction types (e.g. cement) combined with land and house ownership were recorded and used as indicators of wealth.

### 2.3 Material Style of Life

**Figure 2:**
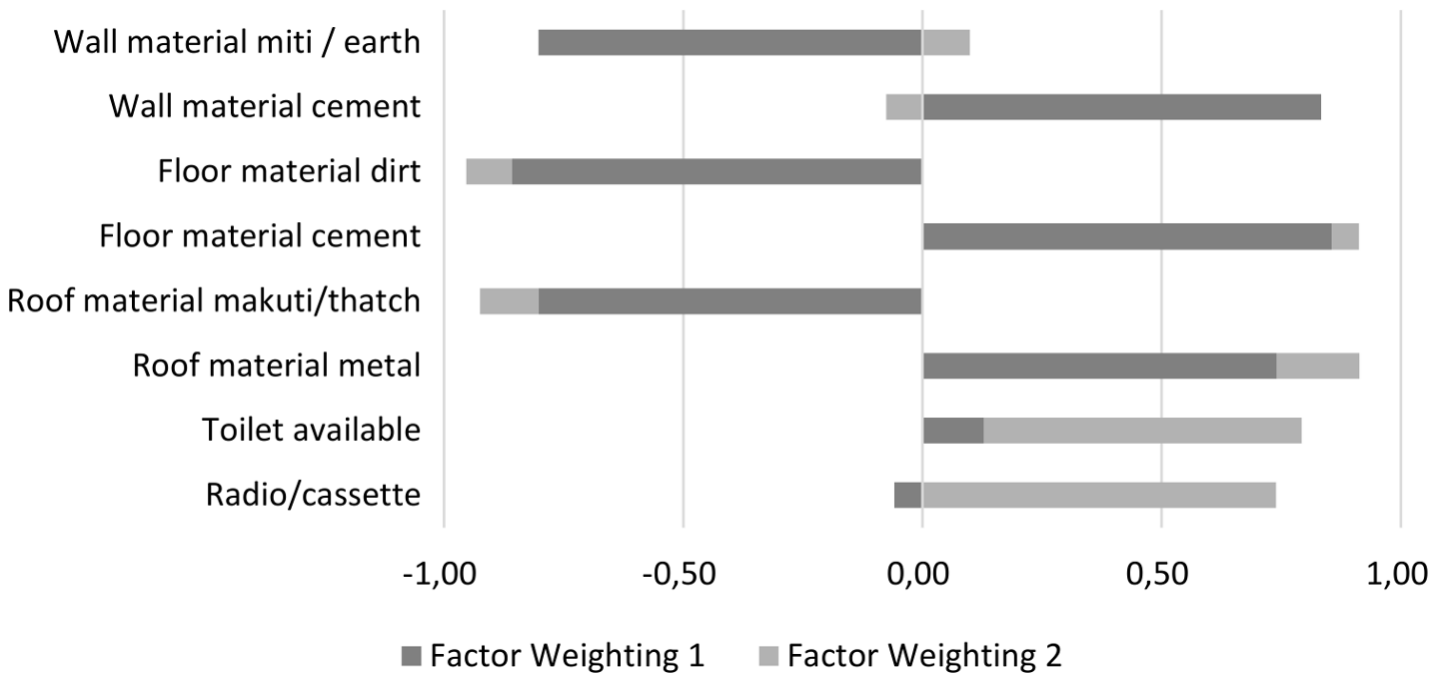
Factor weightings following Varimax Rotation, values indicate contribution of factors (if present) to overall Material Style of Life score, n = 299.

A material style of life (MSL) unitary measure was calculated on the basis of rotated component weightings obtained using a factor analysis with Varimax rotation applied to all data for presence or absence of key assets [17]. Key assets examined were all household possessions and utilities examined in interviews. These were equivalent to the selection in previous analysis of fisher village survey data as detailed by Cinner et al. [17] with the additions made in the current study including land ownership, ownership of mobile phones, availability of water services and availability of electricity. Assets with weightings between 0.3 or −0.3 in first and second components after Varimax rotation were excluded. Remaining factor weightings for all individuals were summed to create a compact MSL score for each respondent household (Figure 2).

### 2.4 Statistical analysis and ordinal regression modelling

A cumulative link model [18] was fitted to the data with responses to the ordinal variable ‘perception of rule-breaking’ (for rules controlling fishing) as the dependent variable with four ordered response categories as follows:

4 - Yes, most people break the rules
3 - Yes, some people break them
2 - Yes, but very few people break them
1 – No, no-one breaks them

**Figure 3:**
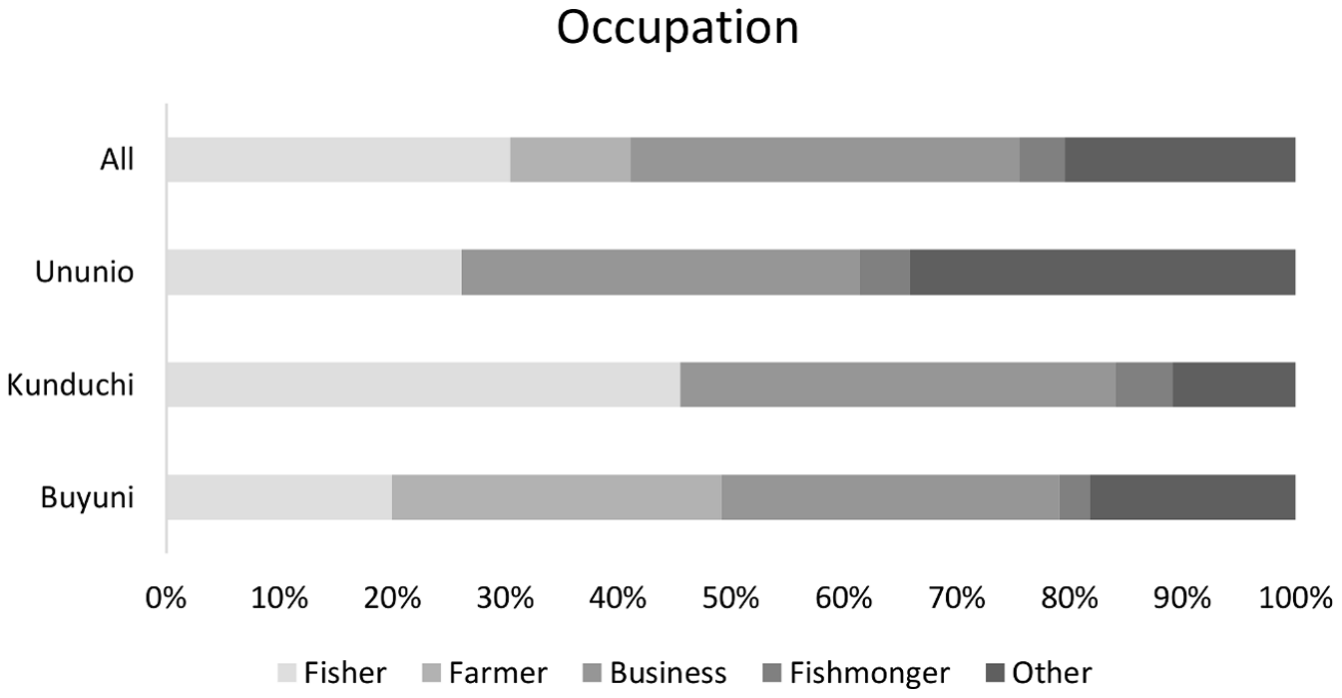
Distribution of occupations by village and all respondents, n = 292.

**Figure 4:**
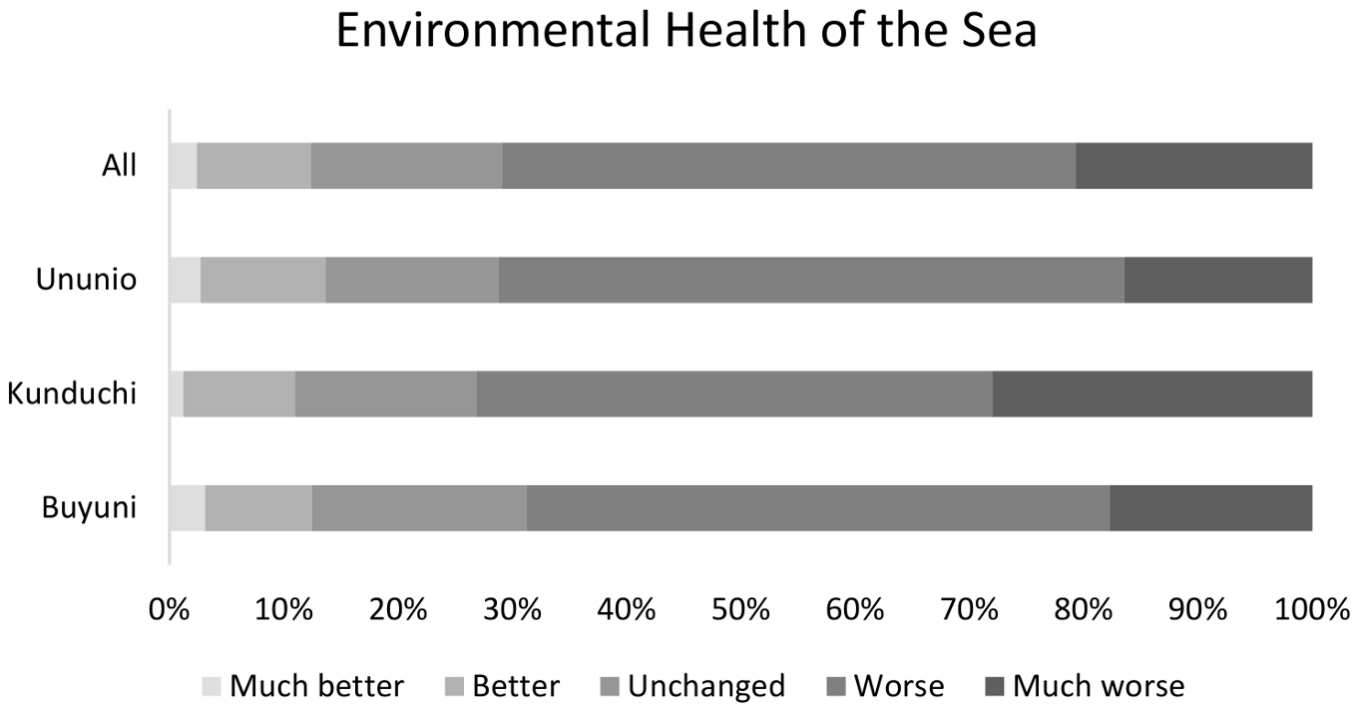
Distribution of individual response by village and all respondents for estimate of environmental health of the sea, n = 250.

The following independent variables were selected following a critical appraisal of relevant literature and included in the cumulative link model, constructed using the ordinal package in R Statistical software (Table 1; Figure 3; Figure 4):

**Table 1:** Mean values of demographic data, response variables and key socio-economic indicators by village (n = 299, TZS = Tanzanian Shilling, HH = Household, Env. = environmental).

	Village			
	Buyuni	Kunduchi	Ununio	All villages
Factor/variable	Mean	Mean	Mean	Mean
Age	38 (13)	39 (13)	36 (13)	38 (13)
Years of education	5 (3)	5 (4)	6 (4)	5 (4)
Weekly income per person [23]	13,575 (12,298)	23,902 (22,128)	23,070 (22,515)	19,771 (19,686)
Total household inhabitants	6 (3)	5 (4)	6 (4)	5 (4)
Material Style of Life	−0.3 (1.9)	2.2 (0.8)	1.9 (1.2)	1.2 (1.8)
Social network strength	1(1)	1 (1)	1 (1)	1 (1)

	% responses	% responses	% responses	% responses
Respondent - Female	39.1	53.5	52.3	47.8%
HH main income fishery	35.2	68.1	46.5	49.5%
HH main income other	64.8	31.9	53.5	50.5%
Fishing reliable income? - yes	73.2	69.2	61.9	68.4
Immigrant – born elsewhere	55.5	78.2	63.6	65.6

1. Is fishing a reliable source of income? (yes/no)
2. Estimate of environmental health of the sea (much better/better/same/worse/much worse)
3. Social network strength (Rating 1–3 depending on participation/decision-making in community)
4. Material style of life (MSL)
5. Weekly income per person in household (Tanzanian Shillings - TZS)
6. Occupation (fisher, farmer, businessperson, fishmonger, other)
7. Years of formal education (years)
8. Age (years)
9. Gender (male/female)
10. Immigrant (yes/no)

In order to compare the perceptions of fishers with that of the community as a whole, a separate cumulative link model was constructed incorporating only the responses of commercially active fishers (those who derive income from their catch) with the same variables excluding the variable Occupation.

Each ordinal regression model was refined by removal of the variable with the highest p-value (non-significant variables) stepwise and by refitting the model accordingly. The empirical identifiability (goodness of definition) was determined on the basis of the condition number of the Hessian; numbers below 10^4^ were considered an acceptable level of definition.

Where applicable data were tested for normality and homogeneity of variances using Shapiro-Wilk and Levene's test respectively before application of parametric statistical tests. An independent samples t-test was applied to compare means of rule awareness between fishers and non-fishers.

## Results

The following results section begins by establishing the level of dependence on marine resources and socio-economic status of the interviewed villages. Comparative awareness of rules controlling fishing and respondents' perceptions on the level of rule-breaking amongst community members are then introduced. Finally an ordinal regression model reveals how explanatory variable(s) retained in the model explain levels of perceived rule-breaking.

### 3.1 Marine resource dependence and socioeconomic status of communities

All villages exhibited high dependence on marine and coastal resources, with between 35.2% (Buyuni) and 68.1% (Kunduchi) of households in each village ranking fishing or fish marketing as their main income source (Table 1; Figure 3). Respondents in peri-urban villages Kunduchi (TZS23,902±22,128 SD) and Ununio (TZS 23,070±22,515 SD), reported higher per capita income than the rural villagers of Buyuni (TZS 13,575±12,298SD) however values did not differ significantly. Peri-urban villages had more developed infrastructure – piped water and electricity - due to their proximity to urban networks (Table 1). Respondents from Ununio had the highest mean number of years of education (6.07±3.9 SD) and social network (measured as combined score for participation in groups and involvement in decisions at group and village level) strength score (1.1±1.0 SD). The majority of respondents (68.4%) felt that fishing was a reliable source of income. More than half of respondents (65.6%) were not born in the village they were currently living in and 70.9% of respondents felt that the environmental health of the sea was worse or much worse than in previous years (Figure 4).

### 3.2 Awareness of rules controlling fishing activity

The majority (86.0%) of respondents described at least one rule controlling fishing activity (Table 2). In open and multiple response questioning, fishers described significantly (t = 3.5, df = 297, p<0.01) more specific rules on average (mean = 1.7±0.1 SE) than non-fishers (mean = 1.4±0.1 SE). The most common rule named by all respondents was the ban on use of explosives in fishing (136 mentions/29.7% of total mentions) followed by need for an appropriate fishing license (105 mentions/22.9% of total mentions) for artisanal and commercial fisheries and limits to net mesh size for net fishing (81 mentions/17.7% of total mentions ). Bans on fishing with poisons such as cyanide (13 mentions/2.8% of total mentions) and the recent ban on beach seine fishing methods (13 mentions/2.8% of total mentions) were mentioned least frequently (Table 2).

**Table 2:** Rule awareness (n = 299), rules mentioned/described (n = 299) and respondent perceptions of rule-breaking (n = 222).

Variable	Response category	No. of responses (n)	% responses
Number of rules described	0	42	14.0
	1	85	28.4
	2	147	49.2
	3	21	7.0
	4	4	1.3
Rule description/mentions	No explosives	136	29.7
	Licensing	105	22.9
	Net mesh limits	81	17.7
	No spears/guns	19	4.1
	No beach seine	13	2.8
	Equipment bans	18	3.9
	Fish size limit	19	4.1
	No poison fishing	13	2.8
	Methods bans	17	3.7
	Others	37	8.1
Do people break fishing rules?	Yes, most of them	33	14.9
	Yes, some	70	31.5
	Yes, but very few	91	41.0
	No, no-one	28	12.6

### 3.3 Perceptions on breaking of rules controlling fishing activity

When asked whether people break the afore-mentioned rules, 41.0% of respondents answered that ‘only very few people or individuals’ break the rules, 31.5% of respondents answered that ‘some people break the rules’, 14.9% answered that ‘most people break the rules’ and the remaining 12.6% responded that ‘no-one breaks fishery rules and regulations’ (Table 2). A total of 77 interviewees or 26% of total interviewees were not willing or able to provide a response to this question.

### 3.4 Ordinal regression modelling

Stepwise removal of explanatory variables to refine the ordinal regression model resulted in a final model - for all respondents -which retained three significant explanatory variables:

1. Estimate of environmental health of the sea (much better/better/same/worse/much worse)
2. Immigrant (yes/no)
3. Gender (male/female)

Respondent's estimate of environmental health of the sea exhibited the greatest effect size and significance in the final ordinal regression model (Table 3). The final model predicts perception of rule-breaking to be significantly higher among respondents who; 1) think the sea is less healthy, 2) are immigrants, and 3) are male (Table 3).

**Table 3:** Ordinal regression model (logistic) all respondents' responses to perception of rule-breaking for rules controlling fishing.

(a)	Estimate	Standard Error	z value	Pr(>|z|)
Gender	0.7514	0.2730	2.7521	0.0059218
Environmental health of sea	−0.4894	0.1423	−3.4392	0.0005834
Immigrant status	0.7320	0.2749	2.6633	0.0077369

(b)	Estimate	Standard Error	z value
1|2	−1.5763	0.8295	−1.9004
2|3	0.2417	0.8195	0.2949
3|4	2.5094	0.8457	2.9671

(a) Retained explanatory variables all respondents (n = 201). (b) Threshold coefficients: (log-likelihood: −241.1900, AIC: 494.3801, Condition number of Hessian: 2288.399).

Stepwise removal of explanatory variables to refine the second ordinal regression model, using responses of fishers only (100% male respondents), resulted in a final model - for all respondents - which retained two significant explanatory variables:

1. Estimate of environmental health of the sea (much better/better/same/worse/much worse)
2. Immigrant (yes/no)

The results of the final and refined model predicts perception of rule-breaking to be significantly higher among immigrant fishers and those fishers who think the sea is less healthy (Table 4).

**Table 4:** Ordinal regression model (logistic) fishers' responses to perception of rule-breaking for rules controlling fishing.

(a)	Estimate	Std. Error	z value	Pr(>|z|)
Immigrant status	1.4543	0.4624	3.1453	0.0016591
Environmental health of sea	−0.5250	0.2121	−2.4749	0.0133282

(b)	Estimate	Standard Error	z value
1|2	−2.0255	1.0790	−1.8772
2|3	0.3114	1.0545	0.2953
3|4	2.6657	1.1117	2.3979

(a) Retained explanatory variables fishers (n = 80). (b) Threshold coefficients: (log-likelihood: −89.85624, AIC: 189.7125, Condition number of Hessian: 1294.610).

## Discussion

The current research identifies for the first time a statistically significant linkage between increased individual perceptions of fisheries rule-breaking and individual perceptions of worsening marine environmental health, based on empirical evidence. The findings are valuable in identifying this relationship, dependent variables and perceptions that influence resource users' environmental problem awareness and thus may influence their behavioural patterns. This information about natural resource health, perceptions and the way in which this knowledge may influence decision-making can be used to improve management and policy formulation. This can result in targeted individuals being more likely to support rules, especially if they are tailored and explained within the context of local priorities derived from empirical data that local people were involved in providing and can relate to.

With governments around the world increasing implementation of fishery and marine management measures as conservation tools, this study is timely for policy advisers in offering advice on what variables should be determined to understand the likely efficacy of rules and measures controlling stakeholders. We predict that this information, if considered and built upon, will help the formulation of rules that are more likely to gain support and achieve greater compliance among communities socially and economically dependent on vulnerable marine resources such as over-exploited fisheries. Given the limited resources and capacity available for enforcement, a targeted approach is more likely to be successful in reconciling conservation with sustainable livelihoods and, more generally, marine management goals.

Coastal villages in this study in the United Republic of Tanzania, as in many other parts of the developing world, are still heavily dependent on marine resources for income generation and food security [19]. [19] Most respondents believe that the health of this pivotal marine resource is worsening and while most are aware of rules controlling fishing activity, nearly half of those interviewed perceive high levels of rule-breaking. Ordinal regression modelling revealed immigrant status, gender and individual perceptions of marine resource health are significantly linked to high levels of perceived rule-breaking. Investigation of the level to which these relationships exist in a coastal community could be used to better contextualise stakeholder-led processes that inform on developing rules to support conservation objectives. Significant associations are discussed herein from an implementation perspective, building on prior research relating compliance to problem awareness. Implications for informing future management and enforcement are provided.

The distribution of responses regarding perceived ‘environmental health of the sea’ clearly demonstrates recognition of marine ecosystem degradation and constitutes problem awareness [14] at the individual level among more than seventy percent of respondents. In addition to apparent problem awareness, the ordinal regression analysis applied reveals a significant linkage between awareness of this environmental problem and respondents' perception of increased levels of rule-breaking. The current study does not test willingness to comply or acceptability of rule-breaking among respondents *per se*. However, applying existing propositions from the literature regarding problem awareness at an individual and societal level, the environmental problem awareness observed has been shown to be significantly linked to perceptions of rule-breaking, and is likely to have a strong positive effect on longer-term decisions on compliance [13]. Amongst individual respondents who are fishers, perception of marine environmental degradation and its linkage to rule-breaking constitutes a positive opportunity for increased compliance dependent on personal conviction of the validity of rules [4], [9], [13]. Equally, those fishers who see deteriorating marine health as a threat to their livelihoods have an individual incentive to decide to comply based on egoistic value threat (sensu Stern [14]). These significant variables can be applied by policy-makers to tailor and contextualise rules to appeal to these perceptions in a socially and economically context-specific relevant setting thus facilitating communication of the problems and possible solutions.

In the current study the belief that marine health is worsening and the linkage between negative marine health perceptions and perceived high levels of rule-breaking are shared by the majority of fishers and non-fisher members of their communities. Thus, while individual fishers recognise threats directly affecting them, the question arises as to whether they will also develop a moral sense of duty to comply in order to protect fishing/coastal community well-being (social-altruistic values) and the environment (biospheric values) [14]. A moral sense of duty to comply on the basis of social-altruistic values can be augmented by peer-group expectation and solidarity, however it is unclear to what degree fishers and non-fishers publicly voice their concerns regarding marine health or expectations regarding compliance. Sutinen and Kuperan [2] argue those fishers who are sensitive to societal or moral expectations may be motivated to reduce rule-breaking. Even fishers who do not perceive marine health degradation or do not associate it with rule-breaking at an individual level, may be influenced by this perception at a community level if held widely enough. Policy-makers and managers tailoring rules and management measures within a community context can thus effectively exploit community expectations and problem awareness to improve compliance. This does not, however, imply improvement on the basis of increased legitimacy of the governing body creating those rules [4], [9], [20].

Without internal or external drivers to commit fishers to compliance, enforcement is unlikely to be economically viable or effective [4], [6], [13]. While some targeted enforcement may be effective this can only be carried out if the underlying socio-economic factors determining fishers' decisions to comply are understood and addressed [2], [3]. The awareness of problems identified by respondents in the current study highlights an opportunity at local and wider contexts to influence future compliance through what Sutinen et al 1990 termed “greater use of the non-coercive factors of compliance (social pressure, self interest, inducement, and obligation)” by framing problems using context-specific management measure options on how they could be tackled. These non-coercive factors are made more powerful, wide-reaching and effective through increased community and individual participation and communication of perceptions. In the specific context of the area studied this would involve increasing community group activities, particularly those with a marine awareness focus and/or with an opportunity for members to express their concerns about or awareness or marine health deterioration. Non-coercive factors benefit also from increased knowledge of the rules and their environmental objectives through public awareness activities, and from activities to cement perceptions of the causal relationship between rule-breaking and marine environmental degradation [3]. In the context of the current study area, public awareness campaigns highlighting key rules and indicating effects of the most common forbidden marine activities on marine health would be valuable.

There is little available literature to explore the significance of immigrant status and gender in explaining rule-breaking the final ordinal regression model presented herein. Immigrant status has previously been linked to increased negativity of perceptions of personal health and well-being but has been shown not to increase rule-breaking behaviour significantly amongst minority immigrants in a (developed) urban context [21], [22]. While our definition of “immigrant”, not born in this village, is broad in the current study, immigrants were significantly more likely to perceive rule-breaking compared with individuals born in the village. It is possible that immigrants experience less sense of social cohesion and thus less compulsion to not admit rule-breaking by other community members, if they sense a lower social cohesion within their fishing groups they may also not adhere to the usual lack of self-enforcement among fishers [8]. Conversely, however, if immigrants are poorly integrated into coastal communities, then there may be a decreased value of social pressure and obligation to adhere to rules as non-coercive factors in driving compliance [1 2006]. The results indicate a clear need for future research to determine the underlying differences in perceptions of rule-breaking among immigrants. Our inferences remain equivocal about why women were significantly less likely to perceive rule-breaking when compared to men, however, this may be best or partly explained by individual experience of fishing being extremely low among female respondents. Nonetheless, gender differences in perceptions towards rule-breaking warrants further investigation especially in terms of how knowledge on the way information is passed between different generations and how this might be used by women to influence compliance behaviour within their households and communities.

## Conclusions

The current study complements existing knowledge identifying a chain of linkages between environmental awareness and environmental compliance. It does so by relating, for the first time, individual perceptions of rule-breaking with perceived degradation of the marine environment. Empirical data analysis points out these key linkages, providing insights into complex compliance processes. This study shows how problem awareness and orientation can provide a focal point for policy makers to inform development of more effective marine management measures. The authors recommend wider application of socio-economic indicators to aid development of rules that are context-specific for those targeted and most likely to be impacted. This may help to identify self-regulation and compliance measures that can be adopted following promotion of existing non-coercive factors. Increasing awareness of environmental health through education programs to target wider understanding of linkages between marine health and compliance, combined with promoting social environmental responsibility, are actions recommended as part of longer term management strategies aimed at supporting sustainable use of marine resources.
